# Evaluation of drug prescribing patterns and therapeutic drug monitoring practice using electronic medical records

**DOI:** 10.1038/s41598-022-25794-y

**Published:** 2022-12-09

**Authors:** Sangmi Lee, Seonghae Yoon, In-Jin Jang

**Affiliations:** 1 Department of Clinical Pharmacology and Therapeutics, Seoul National University College of Medicine and Hospital, Seoul, Korea; 2 Department of Clinical Pharmacology and Therapeutics, Seoul National University College of Medicine and Bundang Hospital, 82, Gumi-Ro 173 Beon-Gil, Bundang-Gu, Seonanam-Si, 13620 Republic of Korea; 3 Integrated Major in Innovative Medical Science, Seoul National University Graduate School, Seoul, Korea

**Keywords:** Health care, Medical research

## Abstract

Therapeutic drug monitoring (TDM) is performed for drugs with narrow therapeutic indices. At Seoul National University Hospital (SNUH) and Seoul National University Bundang Hospital (SNUBH), TDM services are provided for various drugs such as antibiotics and antiepileptics. This study aimed to identify prescription patterns over time using electronic medical records and analyze their relationship with TDM practice. Data were collected from a clinical data warehouse from 2007 to 2020, and the number of patients, total number of drug administration days, serum level tests, and TDM were calculated. The ratio was calculated as the number of serum level tests or TDM to the total number of drug administration days. The study included 136,427 and 162,927 patients from SNUH and SNUBH who were prescribed 11 specified drugs. Each drug showed different prescription patterns over time, and the serum level test and TDM also changed with prescription pattern changes. Serum level test or TDM of antibiotics was frequently used compared to antiepileptics. As some drugs’ usage and test for drugs have decreased newly developed drugs are replacing old drugs. It is recommended that TDM services include these new drugs as well for an effective and safe therapy.

## Introduction

Therapeutic drug monitoring (TDM) of narrow therapeutic index (NTI) drugs has been conducted for optimal pharmacotherapy since the 1970s^1,2^. NTI drugs have a narrow range between the effective doses and those with toxic effects^3^. Thus, small changes in systemic concentrations can lead to considerable changes in drug effect or toxicity^3–5^. Aminoglycoside, lithium, digoxin, phenytoin, and carbamazepine are representatives of NTI drugs^3^. By performing TDM, the dosage and usage can be adjusted so that the drug concentration is within the therapeutic window to maximize the therapeutic effect and minimize adverse events^1,4,6^.

The drug concentration of vancomycin, a drug that requires TDM, has been evaluated and an optimal dosage regimen can be suggested^7^. Trough concentration has been widely used, and recently, area-under the curve of concentration (AUC) has also been recommended for vancomycin TDM^8–10^. For aminoglycoside TDM, peak and trough concentrations are used because toxicity is related to peak level and efficacy is related to trough level^11^. Most psychiatric drugs are taken for a long period of time, and some require evaluation of drug concentrations. There was a study that evaluated the prescription pattern and TDM of psychiatric drugs and they compared the doses and serum concentrations between the treatment group in a high-security psychiatric unit and the control group^12^. According to the study, TDM does not have a great impact on the prescribed doses of psychiatric drugs, but it can be a tool to confirm patient adherence^12^.

Electronic medical record (EMR) data have been widely used in retrospective studies^13^. There have been several studies on infections in the bloodstream, errors in antibiotic prescriptions, and monitoring of antibiotic usage using data from the clinical data warehouse (CDW)^14,15^. TDM for several drugs has been conducted for more than a decade at Seoul National University Hospital (SNUH) and Seoul National University Bundang Hospital (SNUBH). The drugs studied include vancomycin, amikacin, gentamicin, tobramycin, digoxin, valproate, carbamazepine, phenytoin, phenobarbital, theophylline, and lithium. In this study, we aimed to evaluate drug prescription patterns and analyze their relationship with the practice of TDM of the 11 previously stated drugs using data from CDW.

## Results

### Patient demographics

This study included patients who had been prescribed drugs at least once. There were no significant differences in the average age and sex between SNUH (136,427 patients) and SNUBH (162,927 patients) (Table 1). The average patient age was 40 to 60 years for most drug groups, but for lithium, it was 37.4 years in SNUH and 37.6 years in SNUBH. The average age of patients who were prescribed gentamicin in SNUBH was 39.0 years, while it was 52.3 years in SNUH.

**Table 1:** Demographics of the patient population.

	Seoul National University Hospital			Seoul National University Bundang Hospital		
	Age* (mean ± SD)	Sex		Age* (mean ± SD)	Sex	
		Male (%)	Female (%)		Male (%)	Female (%)
Vancomycin	59.9 ± 17.0	16,660 (58.5)	11,817 (41.5)	58.1 ± 23.2	8995 (51.0)	8655 (49.0)
Amikacin	52.9 ± 20.2	3727 (63.7)	2128 (36.3)	45.5 ± 28.1	3666 (57.4)	2720 (42.6)
Gentamicin	52.3 ± 19.7	12,357 (36.8)	21,238 (63.2)	39.0 ± 28.8	12,936 (52.1)	11,886 (47.9)
Tobramycin	53.7 ± 16.4	4521 (50.4)	4449 (49.6)	49.1 ± 17.7	7183 (37.7)	11,880 (62.3)
Valproate	47.3 ± 18.2	7573 (52.6)	6817 (47.4)	46.9 ± 23.0	9748 (47.0)	10,979 (53.0)
Phenytoin	48.4 ± 17.3	2013 (57.8)	1472 (42.2)	42.7 ± 28.3	1346 (57.7)	985 (42.3)
Carbamazepine	54.4 ± 16.1	7322 (44.4)	9184 (55.6)	52.7 ± 18.0	2110 (41.3)	2999 (58.7)
Phenobarbital	51.6 ± 17.0	1433 (50.6)	1401 (49.4)	40.6 ± 26.5	1004 (43.8)	1288 (56.2)
Digoxin	65.0 ± 13.5	7019 (55.8)	5556 (44.2)	68.0 ± 16.2	4418 (57.5)	3264 (42.5)
Theophylline	64.8 ± 13.2	3014 (55.0)	2465 (45.0)	59.5 ± 21.3	1806 (55.3)	1459 (44.7)
Lithium	37.4 ± 15.9	1849 (43.4)	2412 (56.6)	37.6 ± 15.1	1564 (36.9)	2673 (63.1)

*The age was calculated to average. The ratio was rounded to the first decimal place.

Differences were observed in the sex ratios of some drugs between the two hospitals. In SNUH, among the patients prescribed gentamicin, the proportion of women (63.2%) was higher than that of men (36.8%). In SNUBH, carbamazepine was prescribed more frequently in women (58.7%) than in men (41.3%), and in the case of lithium, the proportion of women (63.1%) was higher than that of men (36.9%).

### Total number of drug administration days, serum level tests, and therapeutic drug monitoring

**Figure 1:**
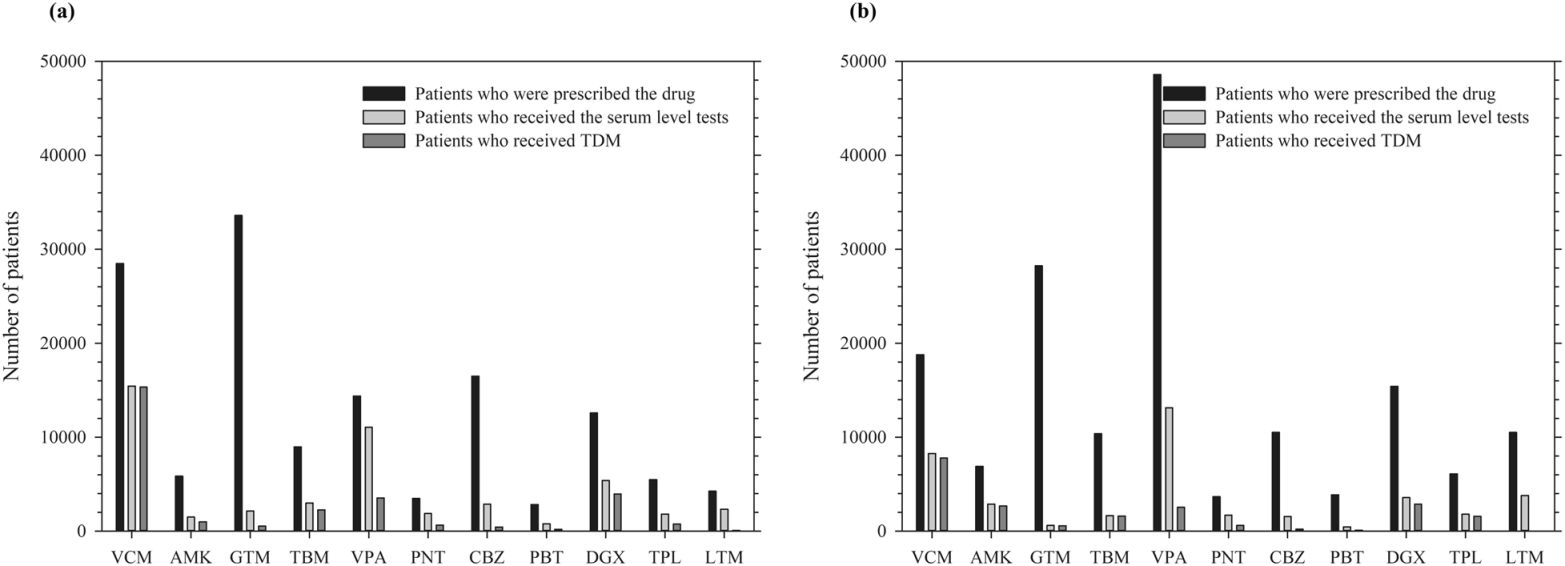
Patients who were prescribed drugs, serum level tests, or therapeutic drug monitoring. (**a**) Number of patients in Seoul National University Hospital and (**b**) Number of patients in Seoul National University Bundang Hospital. Abbreviation: VCM, vancomycin; AMK, amikacin; GTM, gentamicin; TBM, tobramycin; VPA, valproate; CBZ, carbamazepine; PNT, phenytoin; PBT, phenobarbital; DGX, digoxin; TPL, theophylline; LTM, lithium.

IN SNUH and SNUBH, the number of patients who were prescribed each drug and the number of patients who underwent a serum level test or TDM was evaluated (Fig. 1).

**Figure 2:**
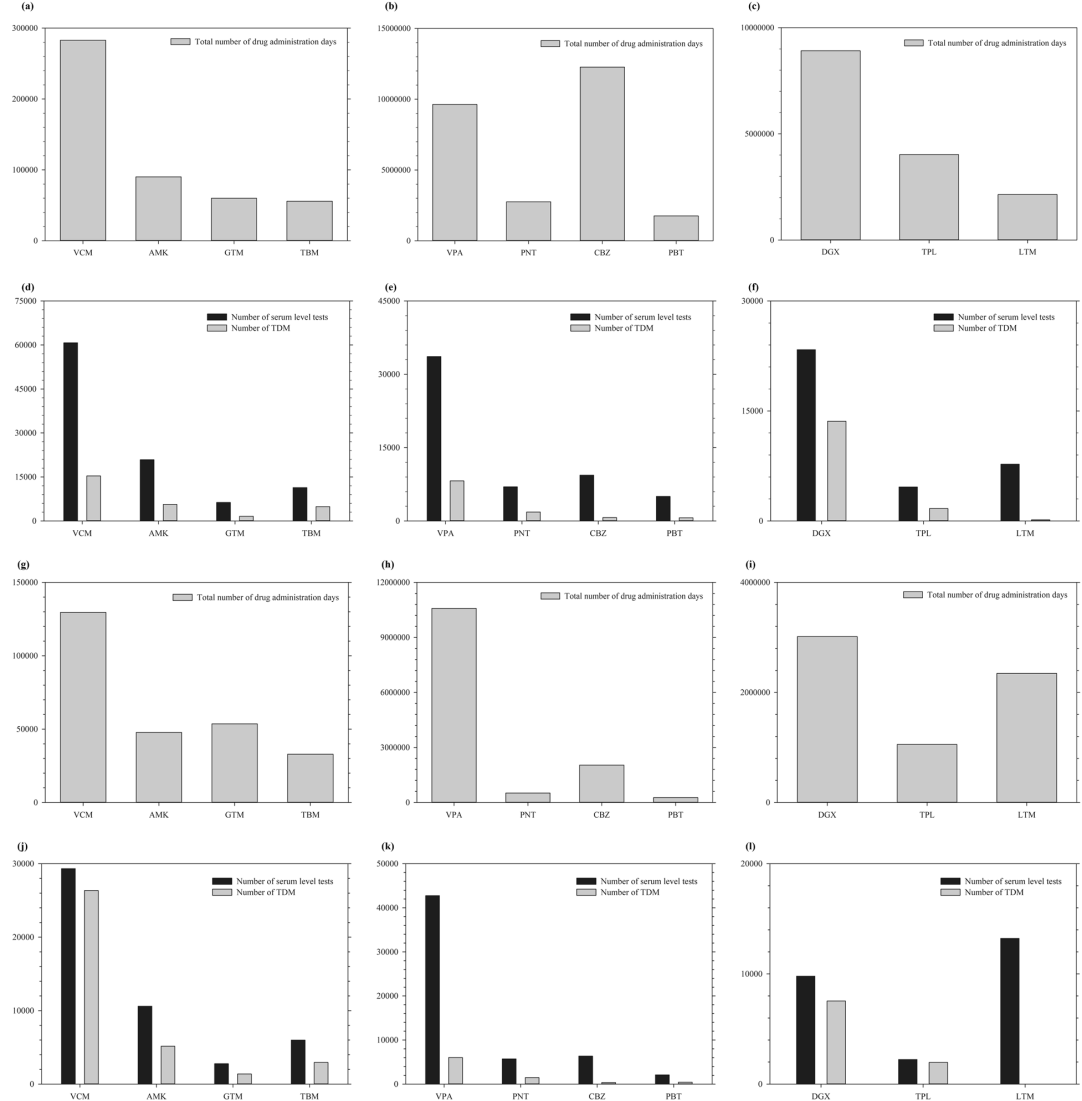
The total number of drug administration days, serum level tests, or therapeutic drug monitoring (**a**), (**g**) Total number of drug administration days of antibiotics; (**b**), (**h**) total number of drug administration days of antiepileptic drugs; (**c**), (**i**) total number of drug administration days of other drugs; (**d**), (**j**) number of serum level tests and therapeutic drug monitoring of antibiotics; (**e**), (**k**) number of serum level tests and therapeutic drug monitoring of antiepileptic drugs; (**f**), (**l**) number of serum level tests and therapeutic drug monitoring of other drugs. Figure (**a**)–(**f**) for Seoul National University Hospital and figure (**g**)–(**l**) for Seoul National University Bundang Hospital. Abbreviation: VCM, vancomycin; AMK, amikacin; GTM, gentamicin; TBM, tobramycin; VPA, valproate; CBZ, carbamazepine; PNT, phenytoin; PBT, phenobarbital; DGX, digoxin; TPL, theophylline; LTM, lithium.

In the case of antibiotics (vancomycin, amikacin, gentamicin, and tobramycin), serum level tests were performed on average approximately once every 6–8 days, and for TDM, once every 16–21 days (Fig. 2, Table 2). Among the antibiotics, vancomycin had the highest total number of drug administration days, serum level tests, and TDM. In the case of antiepileptics (valproate, phenytoin, carbamazepine, and phenobarbital), serum level tests were performed once every 1–2 years on average, but TDM was not performed frequently in either hospital. Carbamazepine was the drug with the highest total number of drug administration days, whereas valproate was the drug with the highest number of serum level tests and TDM in SNUH. In SNUBH, valproate had the highest total number of drug administration days, serum level tests, and TDM. In the case of other drugs (digoxin, theophylline, and lithium), serum level tests were performed once every 2–3 years on average, but TDM was rarely performed in either hospital. TDM for lithium was not performed at SNUBH.

**Table 2:** Total number of drug administration days, number of serum level tests and therapeutic drug monitoring, and average administration days per serum level test or therapeutic drug monitoring.

	Seoul National University Hospital				
	Total number of Drug administration days (day)	Number of serum level tests (N)	Number of TDM (N)	Average drug administration days per serum level test* (day)	Average drug administration days per TDM* (day)
Vancomycin	283,030	60,767	15,326	4.7	18.5
Amikacin	90,148	20,855	5,635	4.3	16.0
Gentamicin	59,899	6295	1,605	9.5	37.3
Tobramycin	55,762	11,384	4,885	4.9	11.4
Valproate	9,632,245	33,631	8,188	286.4	1176.4
Phenytoin	2,754,477	6978	1,818	394.7	1515.1
Carbamazepine	12,271,882	9361	693	1311	17,708.3
Phenobarbital	1,760,982	5015	650	351.1	2709.2
Digoxin	8,915,373	23,360	13,610	381.7	655.1
Theophylline	4,019,776	4625	1,701	869.1	2363.2
Lithium	2,141,764	7731	147	277.0	14,569.8

	Seoul National University Bundang Hospital				
	Total number of Drug administration days (day)	Number of serum level tests (N)	Number of TDM (N)	Average drug administration days per serum level test* (day)	Average drug administration days per TDM * (day)
Vancomycin	129,635	29,335	26,346	4.4	4.9
Amikacin	47,723	10,595	5151	4.5	9.3
Gentamicin	53,618	2772	1376	19.3	39
Tobramycin	32,930	5992	2941	5.5	11.2
Valproate	10,585,806	42,750	6022	247.6	1757.9
Phenytoin	514,699	5719	1477	90	348.5
Carbamazepine	2,039,616	6337	361	321.9	5649.9
Phenobarbital	261,700	2091	428	125.2	611.4
Digoxin	3,016,601	9795	7539	308	400.1
Theophylline	1,055,304	2233	1965	472.6	537.1
Lithium	2,346,433	13,229	–	177.4	–

*The ratio was rounded to the first decimal place.

### Comparison of the number of patients, serum level tests, and therapeutic drug monitoring

**Figure 3:**
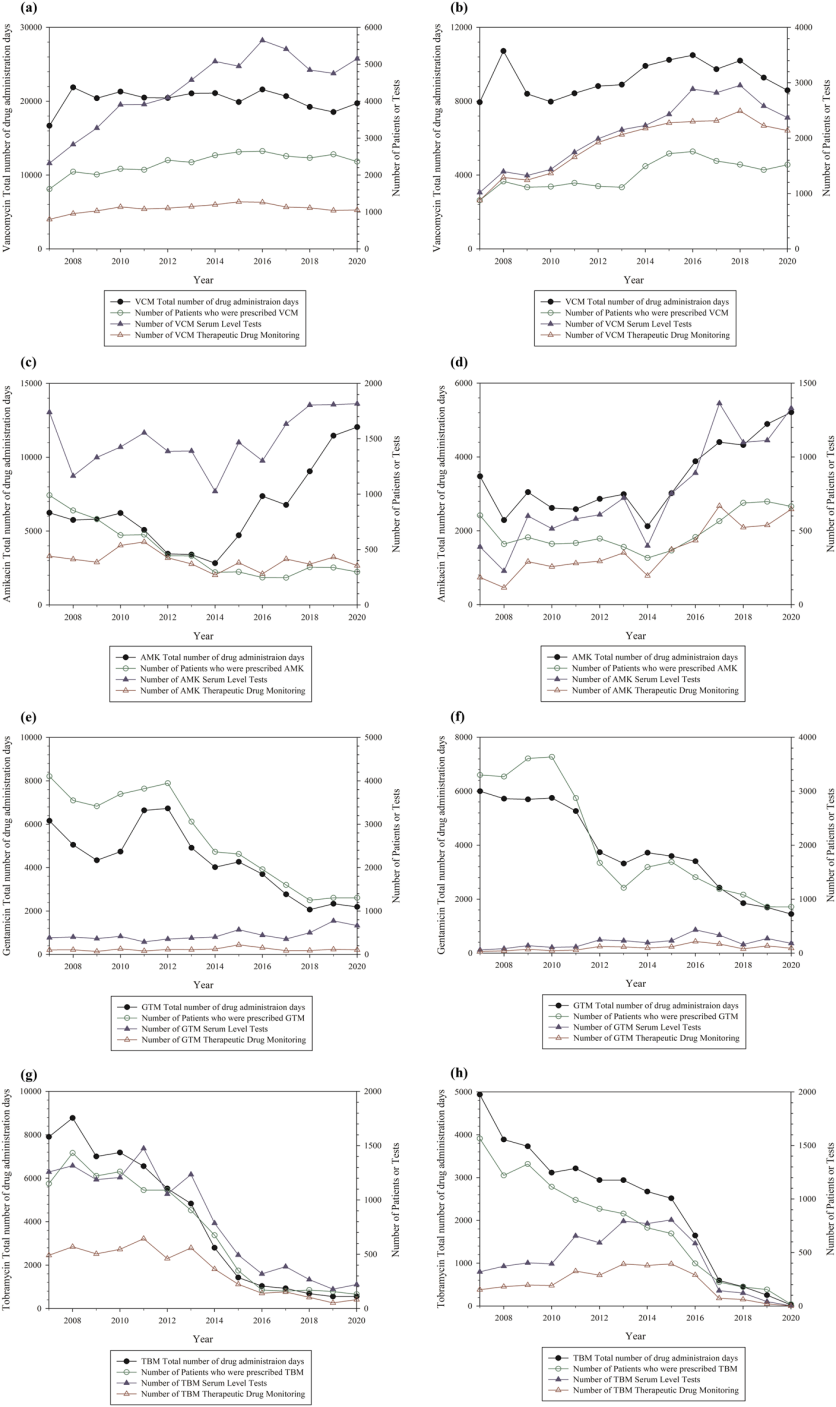
Analysis of the total number of drug administration days and the cases of serum level tests and therapeutic drug monitoring by year in Seoul National University Hospital or Seoul National University Bundang Hospital (Antibiotics: vancomycin, amikacin, gentamicin, and tobramycin). (**a**), (**c**), (**e**), (**g**) for Seoul National University Hospital and (**b**), (**d**), (**f**), (**h**) for Seoul National University Bundang Hospital. The left y-axis represents the total number of drug administration days and the right y-axis represents the number of patients who were prescribed drugs and the number of serum level tests or therapeutic drug monitoring. Abbreviation: VCM, vancomycin; AMK, amikacin; GTM, gentamicin; TBM, tobramycin.

**Figure 4:**
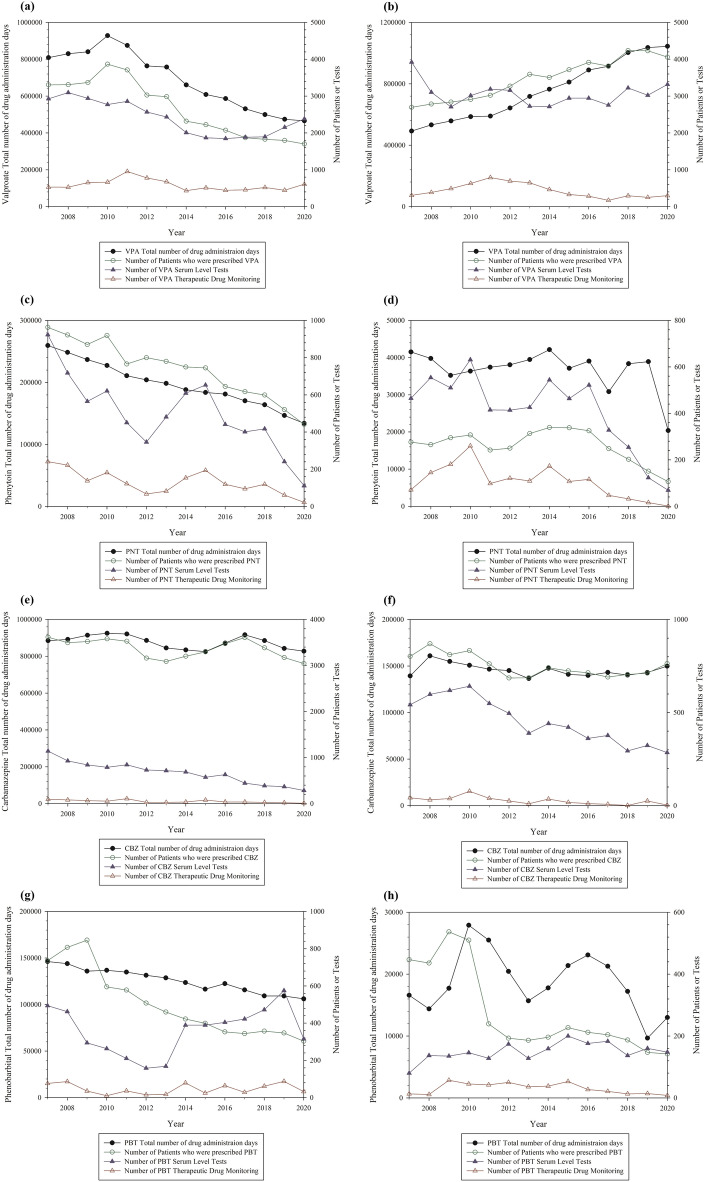
Analysis of the total number of drug administration days and the cases of serum level tests and therapeutic drug monitoring by year in Seoul National University Hospital or Seoul National University Bundang Hospital (Antiepileptic drugs: valproate, phenytoin, carbamazepine, and phenobarbital). (**a**), (**c**), (**e**), (**g**) for Seoul National University Hospital and (**b**), (**d**), (**f**), (**h**) for Seoul National University Bundang Hospital. The left y-axis represents the total number of drug administration days and the right y-axis represents the number of patients who were prescribed drugs and the number of serum level tests or therapeutic drug monitoring. Abbreviation: VPA, valproate; CBZ, carbamazepine; PNT, phenytoin; PBT, phenobarbital.

**Figure 5:**
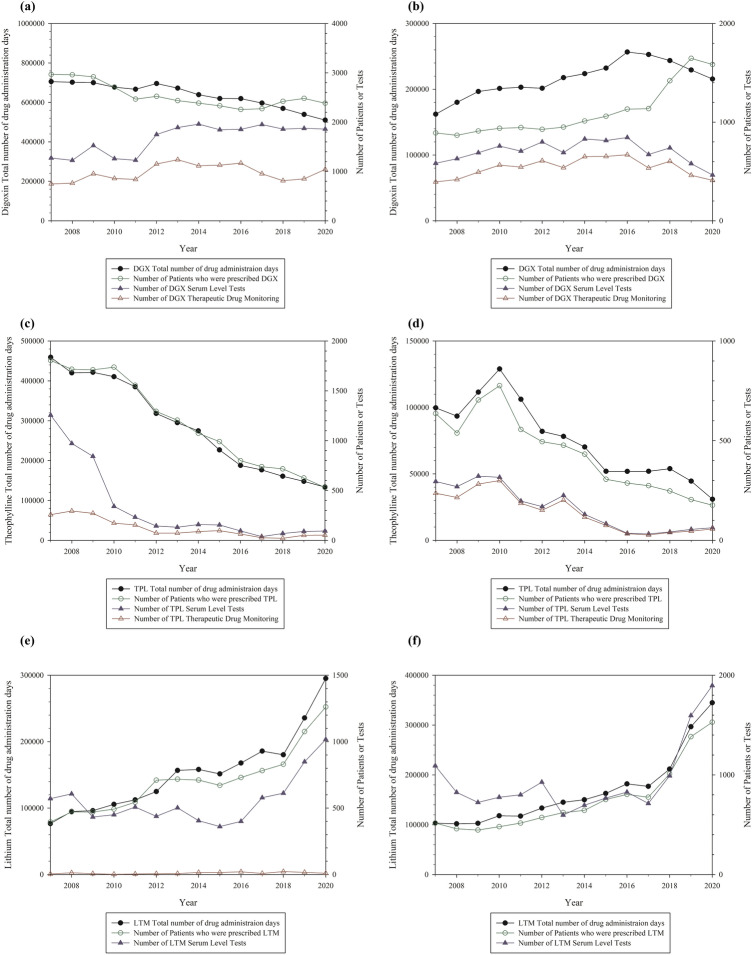
Analysis of the total number of drug administration days and the cases of serum level tests and therapeutic drug monitoring by year in Seoul National University Hospital or Seoul National University Bundang Hospital (Other drugs: valproate, theophylline, and lithium). (**a**), (**c**), (**e**) for Seoul National University Hospital and (**b**), (**d**), (**f**) for Seoul National University Bundang Hospital. The left y-axis represents the total number of drug administration days and the right y-axis represents the number of patients who were prescribed drugs and the number of serum level tests or therapeutic drug monitoring. There were no reports of lithium TDM in Seoul National University Bundang Hospital. Abbreviation: DGX, digoxin; TPL, theophylline; LTM, lithium.

Changes in the number of patients, serum level tests, and TDM over time are shown in Figs. 3, 4, and 5. Among the antibiotics analyzed, vancomycin showed no considerable changes over time in the number of patients, serum level tests, or TDM for 14 years in both hospitals. The number of patients prescribed amikacin has been continuously decreasing in SNUH. However, in SNUBH, the number of patients decreased until 2014 but then increased again, as did the number of serum level tests and TDM. For gentamicin, the total number of drug administration days and number of patients decreased in both hospitals. The number of serum level tests and TDM did not change markedly over time. For tobramycin, the total number of drug administration days and number of serum level tests decreased in SNUH. The number of TDM did not change markedly until 2014 but has decreased since 2014.

**Figure 6:**
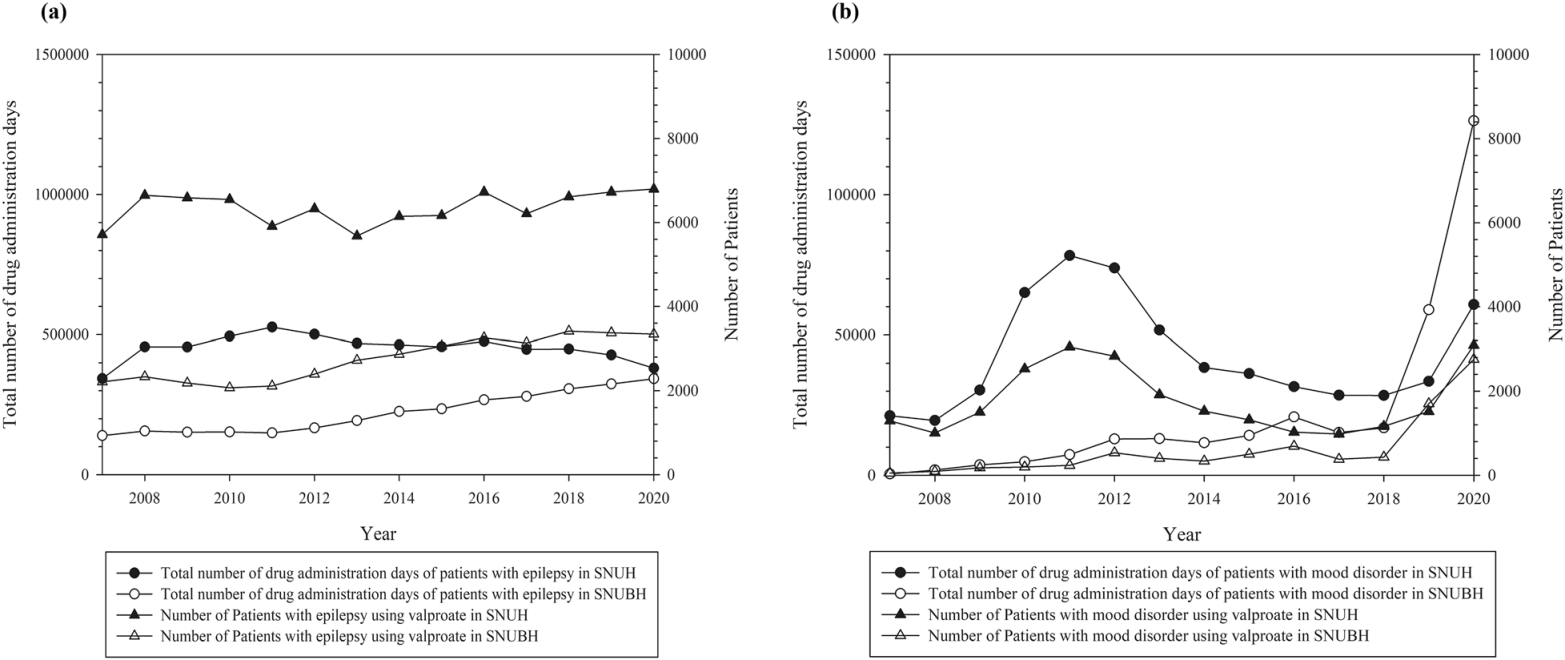
The total number of drug administration days and the number of patients who were prescribed valproate for mood disorder or epilepsy by year in Seoul National University Hospital and Seoul National University Bundang Hospital (**a**) for epilepsy and (**b**) for mood disorder. The left y-axis represents the total number of drug administration days and the right y-axis represents the number of patients who were prescribed valproate.

From the antiepileptics studied (valproate, phenytoin, carbamazepine, and phenobarbital), the valproates showed different trends between the two hospitals. In SNUH, the number of patients who were prescribed valproate and the total number of drug administration days increased by 2010 and decreased thereafter. In contrast, the number of patients and the total number of drug administration days increased in SNUBH. The number of serum level tests and TDM remained unchanged in both hospitals. Additional analysis was conducted to identify trends for two main indications of valproate: epilepsy and mood disorders (Fig. 6). SNUH and SNUBH showed different trends for each valproate indication. In the case of epilepsy, the number of patients and the total number of drug administration days did not change markedly over time in either hospital. In the case of mood disorders, the number of patients and the total number of drug administration days have increased in the past two years in both hospitals, especially in SNUBH. We assumed that these changes influenced the overall pattern of the change in valproate. The number of patients and the total number of drug administration days of phenytoin decreased continuously in SNUH. The number of serum level tests and the TDM of phenytoin have also decreased since 2016. In SNUBH, the number of patients, serum level tests, and TDM have decreased since 2014. Carbamazepine showed no considerable changes in the number of patients or the total number of drug administration days, but the number of serum level tests and TDM has decreased in both hospitals. The number of patients and the total number of days of phenobarbital administration decreased in SNUH, and the number of serum level tests decreased until 2013, then increased again. However, the number of TDMs did not change. In SNUBH, the number of patients showed declining trends, whereas the total number of drug administration days showed some fluctuation. The number of serum level tests and TDM of phenobarbital showed no considerable changes.

Digoxin showed a gradual decrease in the number of patients and the total number of drug administration days. The number of serum level tests showed no considerable changes since 2012. No considerable changes were observed in the number of TDM. In SNUBH, the number of patients has increased continuously, and the total number of drug administration days has decreased since 2015. The number of serum level tests and TDM results showed little change. Theophylline uses decreased steadily in both hospitals; serum level tests and TDM also decreased with a decrease in prescription. For lithium, the number of patients, the total number of drug administration days, and serum levels were analyzed in both hospitals. TDM had rarely been performed in SNUH, and there was no considerable change. Lithium TDM was never performed in SNUBH.

## Discussion

We calculated the number of patients, total number of drug administration days, serum level tests, and TDM of 11 drugs in the SNUH and SNUBH. Through this, we tried to find a relationship between prescription patterns (number of patients and total number of drug administration days) and TDM practice (number of serum level tests and TDM) and to suggest a future direction for TDM services.

For some drugs, the number of TDM changed with an increase or decrease in the number of patients and the total number of drug administration days, but for others, it did not. For gentamicin, antiepileptics (valproate, phenytoin, carbamazepine, phenobarbital), theophylline, and lithium, serum level tests were performed, and then the dosage regimen, in most cases, was adjusted at the discretion of clinicians. Although there is small pharmacodynamic (PD) variability in antibiotics^19^, there could be considerable differences in drug response even at the same drug concentration in the case of antiepileptics or digoxin; thus, the evaluation of effectiveness is important for these drugs^16^. Therefore, some physicians prefer to decide the drug regimen for these drugs only with serum level tests, and the number of TDM cases is small for these drugs compared to other drugs.

It seems that TDM for antibiotics is more frequently performed than for other drugs because these drugs are mostly prescribed to hospitalized patients. TDM for vancomycin is known to prevent adverse events, such as nephrotoxicity or ototoxicity^7^. Vancomycin showed no considerable difference in the number of patients, total number of drug administration days, serum level tests, and TDM in this study. This might be because there was no change in the use of empirical antibiotics, and the rate of MRSA infection was similar or slightly decreased^17–19^. Prescription patterns for tobramycin and amikacin changed around 2014 in both hospitals, which was presumed to be due to changes in drug recommendations in clinical practice. Before 2014, a combination of piperacillin-tazobactam and tobramycin was recommended as empirical antibiotics for the first neutropenia with an unknown infection site and causative agent^20,21^. However, due to the discontinuation of the supply of piperacillin-tazobactam in 2014, the empirical antibiotic recommendations were changed as follows: piperacillin-tazobactam alone or in combination with amikacin at SNUH and a combination of ceftizoxime and amikacin in SNUBH were recommended regimens^22,23^. It appears as if these changes in practice also influenced an increase in amikacin usage. Although there were no changes in the drug usage recommendations for gentamicin, its usage continuously decreased. Gentamicin is rarely prescribed, except for infective endocarditis, as the number of referrals and consultations to the Department of Infectious Diseases has increased^24^.

Antiepileptic drugs (AEDs) are mainly prescribed to control seizures. Because the antiepileptics have large interindividual variability, sometimes patients could have a drug concentration not within the known therapeutic range^16^. Due to differences in the effectiveness of drugs between individuals even at the same drug concentrations, some clinicians prefer to evaluate drug efficacy and control the dosage for each patient rather than depending on TDM consultation. At SNUH, the number of serum level tests and TDM of valproate decreased as drug use decreased. Increased use of levetiracetam, lacosamide, and topiramate could be one of the reasons for the decrease in valproate use. The number of patients and the total number of drug administration days increased, whereas the number of serum level tests and TDM showed no considerable change in SNUBH. While the number of patients prescribed valproate for epilepsy did not change markedly, the number of patients in SNUBH showed a steady increase. On the other hand, the number of patients with mood disorders has shown an increasing trend since 2018, and this trend was more evident in SNUBH. Despite the increase in the number of patients, the number of serum tests and TDM remained similar. In the case of phenytoin, its usage decreased continuously because it was replaced with other drugs, such as lacosamide. Although carbamazepine is being replaced by oxcarbazepine in epilepsy patients, it is frequently prescribed in patients with neuralgia^25,26^. However, serum level tests or TDM are rarely performed in these patients because a low dose of carbamazepine is prescribed for the indication.

Digoxin is used to treat heart failure and atrial fibrillation, and patients need to take the drug for a long time^27^. Thus it seems that serum level tests and TDM are performed approximately once every 1–2 years to check if the drug concentrations are within the therapeutic range. For Theophylline, the number of patients, total number of drug administration days, serum level tests, and TDM decreased in both hospitals. Theophylline is prescribed for patients with respiratory diseases such as chronic obstructive pulmonary disease (COPD) and is administered for a long time^28^. However, since it has adverse reactions that lead to poor medication compliance, it is being replaced by inhaled long-acting beta2 agonists^29^. Lithium is an effective antipsychotic drug for manic disorder or depression^30–32^. The number of patients, total number of drug administration days, and serum level tests increased in both hospitals. Only a few cases of lithium TDM were performed in SNUH and none in SNUBH. Some physicians prefer lithium to valproate for bipolar disorder, and there is a trend for the spectrum of mood disorders to broaden. This phenomenon has led to an increase in lithium use^33^.

As shown so far, it has been confirmed that the number of patients or the total number of drug administration days were different in the SNUH and SNUBH due to various factors; however, this study does have three limitations. First, fewer serum level tests of TDM could be performed in an outpatient setting than in an in-patient. As in-patient and out-patient prescriptions were not analyzed separately, we could not distinguish the differences between the settings. Second, there might be differences in prescription patterns for each indication, and differences for each indication were not analyzed separately. However, valproate, which showed different tendencies in the two hospitals, was further analyzed to determine whether there was a difference according to the indications. Third, bias may occur since this study is a retrospective study compared to a prospective study design. And since data are from tertiary hospitals, it may not be appropriate to apply the results to general hospitals.

In conclusion, we analyzed the prescription patterns of 11 drugs, the number of serum level tests, and TDM at SNUH and SNUBH over 14 years and tried to understand the relationship between them. The changes in the number of TDM differed among the drugs. As new drugs are continuously being developed to replace existing drugs, the use of some drugs and tests for the drugs has decreased. For effective and safe drug therapy, we need to expand TDM services for newly developed drugs.

## Methods

### Ethics approval and study population

The protocol was reviewed and approved by an Institutional Review Board (IRB) of SNUH and SNUBH (SNUH IRB No. H-2103-153-1206, SNUBH IRB No. B-2104-680-402) under the premise that the anonymity of the patient is guaranteed. In consideration of the large number of patients and the fact that this is a retrospective study, obtaining informed consent was exempted by the IRB of SNUH and SNUBH. Our research involving human data has been performed in accordance with the Declaration of Helsinki.

This study included patients who took the following drugs that required TDM: antibiotics (vancomycin, amikacin, gentamicin, tobramycin), antiepileptics (valproate, carbamazepine, phenytoin, and phenobarbital), and other drugs (digoxin, theophylline, and lithium).

### Data collection

Data were extracted from the CDW from January 1, 2007, to December 31, 2020. Patients who were prescribed the drug at least once were included in the study, regardless of age or sex. Ophthalmology formulations or investigational products were excluded, and drugs that did not require TDM were also excluded due to the characteristics of the drug. The capsule formulation of vancomycin does not require TDM because it is used to control infection in the gastrointestinal tract.

In the case of demographics, sex and age were analyzed, and descriptive statistics were presented based on the age at the time of drug description. The total number of drug administration days and the number of prescriptions were calculated for each patient group prescribed the following drugs: vancomycin, amikacin, gentamicin, tobramycin, valproate, carbamazepine, phenytoin, phenobarbital, digoxin, theophylline, and lithium. The total number of drug administration days for each drug was calculated by adding the number of drug administration days for each patient.

The number of serum level tests and cases of TDM counted in patients who were prescribed the drugs. In this study, TDM refers to services including serum level tests and dosage recommendations. For reference, it is necessary to interpret the aminoglycoside data considering that serum level tests are performed twice to check the peak and trough levels in patients who were prescribed aminoglycoside.

### Data analysis

In this study, data were collected for the 11 drugs and classified into three groups. The first was the use of antibiotics, including vancomycin, amikacin, gentamicin, and tobramycin. The second category was antiepileptics, which included valproates, phenytoin, carbamazepine, and phenobarbital. The third category included other drugs, including digoxin, theophylline, and lithium. The total number of drug administration days was calculated to compare prescription patterns because each drug has different usual administration days. To evaluate the relationship between the number of drug administration days and the number of tests, the total number of drug administration days was divided by the number of serum level tests and TDM tests, which represents how often the tests were conducted.

## Supplementary Information

Supplementary Information.

## Data Availability

The datasets generated and analyzed during the current study are not publicly available because data usage was approved only for this study but are available from the corresponding author on reasonable request.
