# Supplementary material for: Evaluation of drug prescribing patterns and therapeutic drug monitoring practice using electronic medical records
Source: Sci Rep. 2022 Dec 9;12:21377. doi: 10.1038/s41598-022-25794-y (PMC9734663; doi:10.1038/s41598-022-25794-y)
Supplement: Supplementary file 1 — Supplementary Information. [file 41598_2022_25794_MOESM1_ESM.docx]

[Supplementary Table]

Supplementary table 1 The number of patients who were prescribed the drug, received serum level tests and therapeutic drug monitoring

| **Institution** | **Seoul National University Hospital** | | | **Seoul National University Bundang Hospital** | | |
| --- | --- | --- | --- | --- | --- | --- |
|  | **Number of patients who were prescribed the drug**  **(N)** | **Number of patients who received serum level test**  **(N)** | **Number of patients who received TDM**  **(N)** | **Number of patients who were prescribed the drug**  **(N)** | **Number of patients who received serum level test**  **(N)** | **Number of patients who received TDM**  **(N)** |
| **Vancomycin** | 28,477 | 15,442 | 15,326 | 18,788 | 8,265 | 7,784 |
| **Amikacin** | 5,855 | 1,509 | 996 | 6,890 | 2,436 | 2,353 |
| **Gentamicin** | 33,595 | 2,137 | 531 | 28,233 | 550 | 529 |
| **Tobramycin** | 8,970 | 2,980 | 2,251 | 10,363 | 1,553 | 1,516 |
| **Valproate** | 14,390 | 11,065 | 3,532 | 48,594 | 21,298 | 2,455 |
| **Phenytoin** | 3,485 | 1,870 | 637 | 3,657 | 2,089 | 560 |
| **Carbamazepine** | 16,506 | 2,875 | 427 | 10,501 | 3,432 | 147 |
| **Phenobarbital** | 2,834 | 769 | 192 | 3,863 | 775 | 92 |
| **Digoxin** | 12,575 | 5,388 | 3,962 | 15,419 | 3,852 | 3,021 |
| **Theophylline** | 5,479 | 1,817 | 756 | 6,108 | 1,097 | 978 |
| **Lithium** | 4,261 | 2,339 | 82 | 10,511 | 6,620 | - |

Supplementary Table 2 Total number of drug administration days, number of serum level tests and therapeutic drug monitoring, and average drug administration days per patients who received serum level tests or therapeutic drug monitoring by year with vancomycin in Seoul National University Hospital

| **Seoul National University Hospital** | | | | | |
| --- | --- | --- | --- | --- | --- |
| **Vancomycin** | **Total number of Drug**  **administration days**  **(day)** | **Number of patients who performed serum level tests**  **(N)** | **Number of patients who performed TDM**  **(N)** | **Average drug administration days per patients who performed serum level test^*^**  **(day)** | **Average drug administration days per patients who performed TDM^*^ (day)** |
| **2007** | 16,681 | 814 | 800 | 20.5 | 20.9 |
| **2008** | 21,878 | 1,040 | 1,030 | 21 | 21.2 |
| **2009** | 20,413 | 1,127 | 1,114 | 18.1 | 18.3 |
| **2010** | 21,301 | 1,264 | 1,260 | 16.9 | 16.9 |
| **2011** | 20,483 | 1,221 | 1,213 | 16.8 | 16.9 |
| **2012** | 20,447 | 1,252 | 1,235 | 16.3 | 16.6 |
| **2013** | 21,064 | 1,284 | 1,275 | 16.4 | 16.5 |
| **2014** | 21,099 | 1,379 | 1,376 | 15.3 | 15.3 |
| **2015** | 19,898 | 1,425 | 1,419 | 14 | 14 |
| **2016** | 21,588 | 1,460 | 1,445 | 14.8 | 14.9 |
| **2017** | 20,680 | 1,328 | 1,314 | 15.6 | 15.7 |
| **2018** | 19,234 | 1,291 | 1,282 | 14.9 | 15 |
| **2019** | 18,542 | 1,235 | 1,223 | 15 | 15.2 |
| **2020** | 19,722 | 1,236 | 1,229 | 16 | 16 |
| The ratio was calculated by dividing the total number of drug administration days by serum level tests or TDM.  ^*^The ratio was rounded to the first decimal place. | | | | | |

Supplementary Table 3 Total number of drug administration days, number of serum level tests and therapeutic drug monitoring, and average drug administration days per patients who received serum level tests or therapeutic drug monitoring by year with amikacin in Seoul National University Hospital

| **Seoul National University Hospital** | | | | | |
| --- | --- | --- | --- | --- | --- |
| **Amikacin** | **Total number of Drug**  **administration days**  **(day)** | **Number of patients who performed serum level tests**  **(N)** | **Number of patients who performed TDM**  **(N)** | **Average drug administration days per patients who performed serum level test^*^**  **(day)** | **Average drug administration days per patients who performed TDM^*^ (day)** |
| **2007** | 6,234 | 683 | 270 | 9.1 | 23.1 |
| **2008** | 5,749 | 429 | 230 | 13.4 | 25 |
| **2009** | 5,804 | 474 | 199 | 12.2 | 29.2 |
| **2010** | 6,218 | 460 | 238 | 13.5 | 26.1 |
| **2011** | 5,072 | 413 | 219 | 12.3 | 23.2 |
| **2012** | 3,456 | 438 | 175 | 7.9 | 19.7 |
| **2013** | 3,400 | 411 | 133 | 8.3 | 25.6 |
| **2014** | 2,822 | 311 | 74 | 9.1 | 38.1 |
| **2015** | 4,710 | 360 | 82 | 13.1 | 57.4 |
| **2016** | 7,364 | 326 | 54 | 22.6 | 136.4 |
| **2017** | 6,771 | 422 | 65 | 16 | 104.2 |
| **2018** | 9,047 | 503 | 77 | 18 | 117.5 |
| **2019** | 11,460 | 461 | 96 | 24.9 | 119.4 |
| **2020** | 12,041 | 458 | 68 | 26.3 | 177.1 |
| The ratio was calculated by dividing the total number of drug administration days by serum level tests or TDM.  ^*^The ratio was rounded to the first decimal place. | | | | | |

Supplementary Table 4 Total number of drug administration days, number of serum level tests and therapeutic drug monitoring, and average drug administration days per patients who received serum level tests or therapeutic drug monitoring by year with gentamicin in Seoul National University Hospital

| **Seoul National University Hospital** | | | | | |
| --- | --- | --- | --- | --- | --- |
| **Gentamicin** | **Total number of Drug**  **administration days**  **(day)** | **Number of patients who performed serum level tests**  **(N)** | **Number of patients who performed TDM**  **(N)** | **Average drug administration days per patients who performed serum level test^*^**  **(day)** | **Average drug administration days per patients who performed TDM^*^ (day)** |
| **2007** | 6,158 | 172 | 59 | 35.8 | 104.4 |
| **2008** | 5,051 | 162 | 57 | 31.2 | 88.6 |
| **2009** | 4,337 | 163 | 36 | 26.6 | 120.5 |
| **2010** | 4,738 | 163 | 50 | 29.1 | 94.8 |
| **2011** | 6,640 | 113 | 32 | 58.8 | 207.5 |
| **2012** | 6,728 | 125 | 35 | 53.8 | 192.2 |
| **2013** | 4,915 | 116 | 33 | 42.4 | 148.9 |
| **2014** | 4,018 | 123 | 40 | 32.7 | 100.5 |
| **2015** | 4,263 | 156 | 54 | 27.3 | 78.9 |
| **2016** | 3,697 | 139 | 44 | 26.6 | 84 |
| **2017** | 2,768 | 131 | 24 | 21.1 | 115.3 |
| **2018** | 2,063 | 141 | 23 | 14.6 | 89.7 |
| **2019** | 2,334 | 247 | 36 | 9.4 | 64.8 |
| **2020** | 2,189 | 217 | 30 | 10.1 | 73 |
| The ratio was calculated by dividing the total number of drug administration days by serum level tests or TDM.  ^*^The ratio was rounded to the first decimal place. | | | | | |

Supplementary Table 5 Total number of drug administration days, number of serum level tests and therapeutic drug monitoring, and average drug administration days per patients who received serum level tests or therapeutic drug monitoring by year with tobramycin in Seoul National University Hospital

| **Seoul National University Hospital** | | | | | |
| --- | --- | --- | --- | --- | --- |
| **Tobramycin** | **Total number of Drug**  **administration days**  **(day)** | **Number of patients who performed serum level tests**  **(N)** | **Number of patients who performed TDM**  **(N)** | **Average drug administration days per patients who performed serum level test^*^**  **(day)** | **Average drug administration days per patients who performed TDM^*^ (day)** |
| **2007** | 7,906 | 408 | 286 | 19.4 | 27.6 |
| **2008** | 8,776 | 427 | 333 | 20.6 | 26.4 |
| **2009** | 7,003 | 380 | 304 | 18.4 | 23 |
| **2010** | 7,186 | 400 | 336 | 18 | 21.4 |
| **2011** | 6,555 | 414 | 316 | 15.8 | 20.7 |
| **2012** | 5,527 | 297 | 235 | 18.6 | 23.5 |
| **2013** | 4,834 | 331 | 285 | 14.6 | 17 |
| **2014** | 2,795 | 196 | 165 | 14.3 | 16.9 |
| **2015** | 1,428 | 123 | 92 | 11.6 | 15.5 |
| **2016** | 1,035 | 53 | 34 | 19.5 | 30.4 |
| **2017** | 926 | 79 | 50 | 11.7 | 18.5 |
| **2018** | 685 | 53 | 32 | 12.9 | 21.4 |
| **2019** | 551 | 39 | 13 | 14.1 | 42.4 |
| **2020** | 555 | 45 | 20 | 12.3 | 27.8 |
| The ratio was calculated by dividing the total number of drug administration days by serum level tests or TDM.  ^*^The ratio was rounded to the first decimal place. | | | | | |

Supplementary Table 6 Total number of drug administration days, number of serum level tests and therapeutic drug monitoring, and average drug administration days per patients who received serum level tests or therapeutic drug monitoring by year with valproate in Seoul National University Hospital

| **Seoul National University Hospital** | | | | | |
| --- | --- | --- | --- | --- | --- |
| **Valproate** | **Total number of Drug**  **administration days**  **(day)** | **Number of patients who performed serum level tests**  **(N)** | **Number of patients who performed TDM**  **(N)** | **Average drug administration days per patients who performed serum level test^*^**  **(day)** | **Average drug administration days per patients who performed TDM^*^ (day)** |
| **2007** | 809,191 | 1,360 | 255 | 595 | 3173.3 |
| **2008** | 830,319 | 1,510 | 247 | 549.9 | 3361.6 |
| **2009** | 840,739 | 1,523 | 363 | 552 | 2316.1 |
| **2010** | 928,547 | 1,554 | 373 | 597.5 | 2489.4 |
| **2011** | 874,992 | 1,651 | 540 | 530 | 1620.4 |
| **2012** | 764,005 | 1,510 | 400 | 506 | 1910 |
| **2013** | 757,685 | 1,381 | 326 | 548.6 | 2324.2 |
| **2014** | 660,889 | 1,200 | 203 | 550.7 | 3255.6 |
| **2015** | 608,692 | 1,133 | 248 | 537.2 | 2454.4 |
| **2016** | 586,642 | 1,140 | 177 | 514.6 | 3314.4 |
| **2017** | 530,976 | 1,046 | 169 | 507.6 | 3141.9 |
| **2018** | 499,623 | 1,137 | 196 | 439.4 | 2549.1 |
| **2019** | 474,193 | 1,100 | 169 | 431.1 | 2805.9 |
| **2020** | 465,752 | 1,211 | 193 | 384.6 | 2413.2 |
| The ratio was calculated by dividing the total number of drug administration days by serum level tests or TDM.  ^*^The ratio was rounded to the first decimal place. | | | | | |

Supplementary Table 7 Total number of drug administration days, number of serum level tests and therapeutic drug monitoring, and average drug administration days per patients who received serum level tests or therapeutic drug monitoring by year with phenytoin in Seoul National University Hospital

| **Seoul National University Hospital** | | | | | |
| --- | --- | --- | --- | --- | --- |
| **Phenytoin** | **Total number of Drug**  **administration days**  **(day)** | **Number of patients who performed serum level tests**  **(N)** | **Number of patients who performed TDM**  **(N)** | **Average drug administration days per patients who performed serum level test^*^**  **(day)** | **Average drug administration days per patients who performed TDM^*^ (day)** |
| **2007** | 259,528 | 362 | 80 | 716.9 | 3244.1 |
| **2008** | 248,562 | 325 | 71 | 764.8 | 3500.9 |
| **2009** | 236,878 | 301 | 56 | 787 | 4230 |
| **2010** | 227,415 | 374 | 98 | 608.1 | 2320.6 |
| **2011** | 210,841 | 265 | 56 | 795.6 | 3765 |
| **2012** | 204,198 | 251 | 32 | 813.5 | 6381.2 |
| **2013** | 198,493 | 263 | 30 | 754.7 | 6616.4 |
| **2014** | 188,236 | 242 | 46 | 777.8 | 4092.1 |
| **2015** | 183,851 | 268 | 55 | 686 | 3342.7 |
| **2016** | 181,220 | 237 | 46 | 764.6 | 3939.6 |
| **2017** | 170,439 | 183 | 36 | 931.4 | 4734.4 |
| **2018** | 164,053 | 196 | 48 | 837 | 3417.8 |
| **2019** | 146,758 | 149 | 22 | 985 | 6670.8 |
| **2020** | 134,005 | 93 | 14 | 1440.9 | 9571.8 |
| The ratio was calculated by dividing the total number of drug administration days by serum level tests or TDM.  ^*^The ratio was rounded to the first decimal place. | | | | | |

Supplementary Table 8 Total number of drug administration days, number of serum level tests and therapeutic drug monitoring, and average drug administration days per patients who received serum level tests or therapeutic drug monitoring by year with carbamazepine in Seoul National University Hospital

| **Seoul National University Hospital** | | | | | |
| --- | --- | --- | --- | --- | --- |
| **Carbamazepine** | **Total number of Drug**  **administration days**  **(day)** | **Number of patients who performed serum level tests**  **(N)** | **Number of patients who performed TDM**  **(N)** | **Average drug administration days per patients who performed serum level test^*^**  **(day)** | **Average drug administration days per patients who performed TDM^*^ (day)** |
| **2007** | 884,569 | 653 | 51 | 1354.6 | 17344.5 |
| **2008** | 891,879 | 579 | 42 | 1540.4 | 21235.2 |
| **2009** | 914,237 | 610 | 36 | 1498.7 | 25395.5 |
| **2010** | 925,004 | 652 | 41 | 1418.7 | 22561.1 |
| **2011** | 921,200 | 693 | 88 | 1329.3 | 10468.2 |
| **2012** | 885,987 | 622 | 24 | 1424.4 | 36916.1 |
| **2013** | 845,284 | 578 | 21 | 1462.4 | 40251.6 |
| **2014** | 834,363 | 547 | 23 | 1525.3 | 36276.7 |
| **2015** | 825,449 | 489 | 63 | 1688 | 13102.4 |
| **2016** | 871,599 | 483 | 22 | 1804.6 | 39618.1 |
| **2017** | 916,491 | 388 | 24 | 2362.1 | 38187.1 |
| **2018** | 885,403 | 346 | 22 | 2559 | 40245.6 |
| **2019** | 842,623 | 318 | 16 | 2649.8 | 52663.9 |
| **2020** | 827,794 | 262 | 8 | 3159.5 | 103474.3 |
| The ratio was calculated by dividing the total number of drug administration days by serum level tests or TDM.  ^*^The ratio was rounded to the first decimal place. | | | | | |

Supplementary Table 9 Total number of drug administration days, number of serum level tests and therapeutic drug monitoring, and average drug administration days per patients who received serum level tests or therapeutic drug monitoring by year with phenobarbital in Seoul National University Hospital

| **Seoul National University Hospital** | | | | | |
| --- | --- | --- | --- | --- | --- |
| **Phenobarbital** | **Total number of Drug**  **administration days**  **(day)** | **Number of patients who performed serum level tests**  **(N)** | **Number of patients who performed TDM**  **(N)** | **Average drug administration days per patients who performed serum level test^*^**  **(day)** | **Average drug administration days per patients who performed TDM^*^ (day)** |
| **2007** | 146,213 | 127 | 17 | 1151.3 | 8600.8 |
| **2008** | 143,998 | 132 | 17 | 1090.9 | 8470.5 |
| **2009** | 135,883 | 132 | 14 | 1029.4 | 9705.9 |
| **2010** | 136,821 | 125 | 8 | 1094.6 | 17102.6 |
| **2011** | 134,883 | 145 | 32 | 930.2 | 4215.1 |
| **2012** | 131,432 | 134 | 12 | 980.8 | 10952.7 |
| **2013** | 128,695 | 124 | 7 | 1037.9 | 18385 |
| **2014** | 123,675 | 128 | 16 | 966.2 | 7729.7 |
| **2015** | 116,546 | 109 | 15 | 1069.2 | 7769.7 |
| **2016** | 122,440 | 117 | 11 | 1046.5 | 11130.9 |
| **2017** | 115,738 | 93 | 11 | 1244.5 | 10521.6 |
| **2018** | 109,302 | 94 | 24 | 1162.8 | 4554.3 |
| **2019** | 109,198 | 111 | 25 | 983.8 | 4367.9 |
| **2020** | 106,158 | 73 | 12 | 1454.2 | 8846.5 |
| The ratio was calculated by dividing the total number of drug administration days by serum level tests or TDM.  ^*^The ratio was rounded to the first decimal place. | | | | | |

Supplementary Table 10 Total number of drug administration days, number of serum level tests and therapeutic drug monitoring, and average drug administration days per patients who received serum level tests or therapeutic drug monitoring by year with digoxin in Seoul National University Hospital

| **Seoul National University Hospital** | | | | | |
| --- | --- | --- | --- | --- | --- |
| **Digoxin** | **Total number of Drug**  **administration days**  **(day)** | **Number of patients who performed serum level tests**  **(N)** | **Number of patients who performed TDM**  **(N)** | **Average drug administration days per patients who performed serum level test^*^**  **(day)** | **Average drug administration days per patients who performed TDM^*^ (day)** |
| **2007** | 705,563 | 556 | 319 | 1269 | 2211.8 |
| **2008** | 702,341 | 548 | 337 | 1281.6 | 2084.1 |
| **2009** | 700,243 | 560 | 336 | 1250.4 | 2084.1 |
| **2010** | 677,506 | 520 | 339 | 1302.9 | 1998.5 |
| **2011** | 666,944 | 522 | 323 | 1277.7 | 2064.8 |
| **2012** | 695,960 | 685 | 402 | 1016 | 1731.2 |
| **2013** | 672,500 | 636 | 357 | 1057.4 | 1883.8 |
| **2014** | 639,155 | 678 | 353 | 942.7 | 1810.6 |
| **2015** | 619,755 | 654 | 378 | 947.6 | 1639.6 |
| **2016** | 619,318 | 663 | 370 | 934.1 | 1673.8 |
| **2017** | 596,901 | 662 | 305 | 901.7 | 1957.1 |
| **2018** | 569,523 | 647 | 280 | 880.3 | 2034 |
| **2019** | 539,311 | 669 | 314 | 806.1 | 1717.6 |
| **2020** | 510,353 | 605 | 320 | 843.6 | 1594.9 |
| The ratio was calculated by dividing the total number of drug administration days by serum level tests or TDM.  ^*^The ratio was rounded to the first decimal place. | | | | | |

Supplementary Table 11 Total number of drug administration days, number of serum level tests and therapeutic drug monitoring, and average drug administration days per patients who received serum level tests or therapeutic drug monitoring by year with theophylline in Seoul National University Hospital

| **Seoul National University Hospital** | | | | | |
| --- | --- | --- | --- | --- | --- |
| **Theophylline** | **Total number of Drug**  **administration days**  **(day)** | **Number of patients who performed serum level tests**  **(N)** | **Number of patients who performed TDM**  **(N)** | **Average drug administration days per patients who performed serum level test^*^**  **(day)** | **Average drug administration days per patients who performed TDM^*^ (day)** |
| **2007** | 459,293 | 866 | 135 | 530.4 | 3402.2 |
| **2008** | 420,158 | 731 | 144 | 574.8 | 2917.8 |
| **2009** | 421,676 | 626 | 128 | 673.6 | 3294.3 |
| **2010** | 410,614 | 254 | 101 | 1616.6 | 4065.5 |
| **2011** | 385,406 | 160 | 95 | 2408.8 | 4056.9 |
| **2012** | 318,480 | 98 | 41 | 3249.8 | 7767.8 |
| **2013** | 295,269 | 58 | 39 | 5090.8 | 7571 |
| **2014** | 274,734 | 95 | 45 | 2891.9 | 6105.2 |
| **2015** | 226,941 | 86 | 33 | 2638.8 | 6877 |
| **2016** | 187,932 | 59 | 33 | 3185.3 | 5694.9 |
| **2017** | 176,804 | 19 | 11 | 9305.5 | 16073.1 |
| **2018** | 160,765 | 44 | 15 | 3653.8 | 10717.7 |
| **2019** | 148,031 | 50 | 27 | 2960.6 | 5482.6 |
| **2020** | 133,673 | 30 | 16 | 4455.8 | 8354.6 |
| The ratio was calculated by dividing the total number of drug administration days by serum level tests or TDM.  ^*^The ratio was rounded to the first decimal place. | | | | | |

Supplementary Table 12 Total number of drug administration days, number of serum level tests and therapeutic drug monitoring, and average drug administration days per patients who received serum level tests or therapeutic drug monitoring by year with lithium in Seoul National University Hospital

| **Seoul National University Hospital** | | | | | |
| --- | --- | --- | --- | --- | --- |
| **Lithium** | **Total number of Drug**  **administration days**  **(day)** | **Number of patients who performed serum level tests**  **(N)** | **Number of patients who performed TDM**  **(N)** | **Average drug administration days per patients who performed serum level test^*^**  **(day)** | **Average drug administration days per patients who performed TDM^*^ (day)** |
| **2007** | 76,568 | 223 | 3 | 343.4 | 25522.7 |
| **2008** | 94,788 | 246 | 5 | 385.3 | 18957.6 |
| **2009** | 96,028 | 226 | 6 | 424.9 | 16004.7 |
| **2010** | 105,595 | 215 | 2 | 491.1 | 52797.5 |
| **2011** | 112,327 | 243 | 4 | 462.3 | 28081.8 |
| **2012** | 124,888 | 226 | 6 | 552.6 | 20814.7 |
| **2013** | 156,808 | 258 | 5 | 607.8 | 31361.6 |
| **2014** | 158,248 | 215 | 9 | 736 | 17583.1 |
| **2015** | 151,514 | 190 | 8 | 797.4 | 18939.3 |
| **2016** | 167,898 | 208 | 13 | 807.2 | 12915.2 |
| **2017** | 185,855 | 292 | 6 | 636.5 | 30975.8 |
| **2018** | 180,271 | 338 | 6 | 533.3 | 30045.2 |
| **2019** | 235,787 | 375 | 10 | 628.8 | 23578.7 |
| **2020** | 295,189 | 523 | 3 | 564.4 | 98396.3 |
| The ratio was calculated by dividing the total number of drug administration days by serum level tests or TDM.  ^*^The ratio was rounded to the first decimal place. | | | | | |

Supplementary Table 13 Total number of drug administration days, number of serum level tests and therapeutic drug monitoring, and average drug administration days per patients who received serum level tests or therapeutic drug monitoring by year with vancomycin in Seoul National University Bundang Hospital

| **Seoul National University Bundang Hospital** | | | | | |
| --- | --- | --- | --- | --- | --- |
| **Vancomycin** | **Total number of Drug**  **administration days**  **(day)** | **Number of patients who performed serum level tests**  **(N)** | **Number of patients who performed TDM**  **(N)** | **Average drug administration days per patients who performed serum level test^*^**  **(day)** | **Average drug administration days per patients who performed TDM^*^ (day)** |
| **2007** | 7,946 | 365 | 346 | 21.8 | 23 |
| **2008** | 10,720 | 493 | 477 | 21.7 | 22.5 |
| **2009** | 8,401 | 454 | 443 | 18.5 | 19 |
| **2010** | 7,975 | 478 | 470 | 16.7 | 17 |
| **2011** | 8,427 | 517 | 503 | 16.3 | 16.8 |
| **2012** | 8,819 | 566 | 556 | 15.6 | 15.9 |
| **2013** | 8,899 | 581 | 574 | 15.3 | 15.5 |
| **2014** | 9,911 | 617 | 609 | 16.1 | 16.3 |
| **2015** | 10,240 | 648 | 629 | 15.8 | 16.3 |
| **2016** | 10,495 | 729 | 637 | 14.4 | 16.5 |
| **2017** | 9,736 | 718 | 626 | 13.6 | 15.6 |
| **2018** | 10,198 | 759 | 678 | 13.4 | 15 |
| **2019** | 9,277 | 688 | 616 | 13.5 | 15.1 |
| **2020** | 8,591 | 652 | 620 | 13.2 | 13.9 |
| The ratio was calculated by dividing the total number of drug administration days by serum level tests or TDM.  ^*^The ratio was rounded to the first decimal place. | | | | | |

Supplementary Table 14 Total number of drug administration days, number of serum level tests and therapeutic drug monitoring, and average drug administration days per patients who received serum level tests or therapeutic drug monitoring by year with amikacin in Seoul National University Bundang Hospital

| **Seoul National University Bundang Hospital** | | | | | |
| --- | --- | --- | --- | --- | --- |
| **Amikacin** | **Total number of Drug**  **administration days**  **(day)** | **Number of patients who performed serum level tests**  **(N)** | **Number of patients who performed TDM**  **(N)** | **Average drug administration days per patients who performed serum level test^*^**  **(day)** | **Average drug administration days per patients who performed TDM^*^ (day)** |
| **2007** | 3,476 | 132 | 122 | 26.3 | 28.5 |
| **2008** | 2,288 | 79 | 78 | 29 | 29.3 |
| **2009** | 3,047 | 143 | 139 | 21.3 | 21.9 |
| **2010** | 2,618 | 142 | 138 | 18.4 | 19 |
| **2011** | 2,584 | 140 | 135 | 18.5 | 19.1 |
| **2012** | 2,864 | 135 | 132 | 21.2 | 21.7 |
| **2013** | 2,991 | 137 | 134 | 21.8 | 22.3 |
| **2014** | 2,122 | 94 | 92 | 22.6 | 23.1 |
| **2015** | 3,018 | 146 | 144 | 20.7 | 21 |
| **2016** | 3,883 | 212 | 205 | 18.3 | 18.9 |
| **2017** | 4,402 | 266 | 257 | 16.5 | 17.1 |
| **2018** | 4,324 | 261 | 250 | 16.6 | 17.3 |
| **2019** | 4,894 | 266 | 255 | 18.4 | 19.2 |
| **2020** | 5,212 | 283 | 272 | 18.4 | 19.2 |
| The ratio was calculated by dividing the total number of drug administration days by serum level tests or TDM.  ^*^The ratio was rounded to the first decimal place. | | | | | |

Supplementary Table 15 Total number of drug administration days, number of serum level tests and therapeutic drug monitoring, and average drug administration days per patients who received serum level tests or therapeutic drug monitoring by year with gentamicin in Seoul National University Bundang Hospital

| **Seoul National University Bundang Hospital** | | | | | |
| --- | --- | --- | --- | --- | --- |
| **Gentamicin** | **Total number of Drug**  **administration days**  **(day)** | **Number of patients who performed serum level tests**  **(N)** | **Number of patients who performed TDM**  **(N)** | **Average drug administration days per patients who performed serum level test^*^**  **(day)** | **Average drug administration days per patients who performed TDM^*^ (day)** |
| **2007** | 6,000 | 18 | 17 | 333.3 | 352.9 |
| **2008** | 5,723 | 30 | 29 | 190.8 | 197.3 |
| **2009** | 5,699 | 29 | 29 | 196.5 | 196.5 |
| **2010** | 5,753 | 29 | 25 | 198.4 | 230.1 |
| **2011** | 5,264 | 25 | 24 | 210.6 | 219.3 |
| **2012** | 3,737 | 44 | 44 | 84.9 | 84.9 |
| **2013** | 3,319 | 42 | 38 | 79 | 87.3 |
| **2014** | 3,720 | 42 | 38 | 88.6 | 97.9 |
| **2015** | 3,595 | 49 | 48 | 73.4 | 74.9 |
| **2016** | 3,402 | 67 | 67 | 50.8 | 50.8 |
| **2017** | 2,421 | 56 | 54 | 43.2 | 44.8 |
| **2018** | 1,848 | 39 | 38 | 47.4 | 48.6 |
| **2019** | 1,690 | 46 | 44 | 36.7 | 38.4 |
| **2020** | 1,447 | 34 | 34 | 42.6 | 42.6 |
| The ratio was calculated by dividing the total number of drug administration days by serum level tests or TDM.  ^*^The ratio was rounded to the first decimal place. | | | | | |

Supplementary Table 16 Total number of drug administration days, number of serum level tests and therapeutic drug monitoring, and average drug administration days per patients who received serum level tests or therapeutic drug monitoring by year with tobramycin in Seoul National University Bundang Hospital

| **Seoul National University Bundang Hospital** | | | | | |
| --- | --- | --- | --- | --- | --- |
| **Tobramycin** | **Total number of Drug**  **administration days**  **(day)** | **Number of patients who performed serum level tests**  **(N)** | **Number of patients who performed TDM**  **(N)** | **Average drug administration days per patients who performed serum level test^*^**  **(day)** | **Average drug administration days per patients who performed TDM^*^ (day)** |
| **2007** | 4,938 | 110 | 106 | 44.9 | 46.6 |
| **2008** | 3,888 | 125 | 124 | 31.1 | 31.4 |
| **2009** | 3,731 | 130 | 125 | 28.7 | 29.8 |
| **2010** | 3,116 | 120 | 115 | 26 | 27.1 |
| **2011** | 3,214 | 168 | 166 | 19.1 | 19.4 |
| **2012** | 2,942 | 155 | 150 | 19 | 19.6 |
| **2013** | 2,941 | 178 | 176 | 16.5 | 16.7 |
| **2014** | 2,674 | 176 | 173 | 15.2 | 15.5 |
| **2015** | 2,517 | 170 | 167 | 14.8 | 15.1 |
| **2016** | 1,645 | 135 | 132 | 12.2 | 12.5 |
| **2017** | 595 | 37 | 36 | 16.1 | 16.5 |
| **2018** | 451 | 36 | 34 | 12.5 | 13.3 |
| **2019** | 254 | 12 | 11 | 21.2 | 23.1 |
| **2020** | 24 | 1 | 1 | 24 | 24 |
| The ratio was calculated by dividing the total number of drug administration days by serum level tests or TDM.  ^*^The ratio was rounded to the first decimal place. | | | | | |

Supplementary Table 17 Total number of drug administration days, number of serum level tests and therapeutic drug monitoring, and average drug administration days per patients who received serum level tests or therapeutic drug monitoring by year with valproate in Seoul National University Bundang Hospital

| **Seoul National University Bundang Hospital** | | | | | |
| --- | --- | --- | --- | --- | --- |
| **Valproate** | **Total number of Drug**  **administration days**  **(day)** | **Number of patients who performed serum level tests**  **(N)** | **Number of patients who performed TDM**  **(N)** | **Average drug administration days per patients who performed serum level test^*^**  **(day)** | **Average drug administration days per patients who performed TDM^*^ (day)** |
| **2007** | 492,391 | 1,573 | 128 | 313 | 3846.8 |
| **2008** | 532,146 | 1,443 | 153 | 368.8 | 3478.1 |
| **2009** | 558,456 | 1,331 | 214 | 419.6 | 2609.6 |
| **2010** | 586,107 | 1,428 | 276 | 410.4 | 2123.6 |
| **2011** | 590,005 | 1,431 | 367 | 412.3 | 1607.6 |
| **2012** | 642,601 | 1,535 | 328 | 418.6 | 1959.1 |
| **2013** | 718,001 | 1,419 | 265 | 506 | 2709.4 |
| **2014** | 765,087 | 1,393 | 145 | 549.2 | 5276.5 |
| **2015** | 811,431 | 1,572 | 116 | 516.2 | 6995.1 |
| **2016** | 890,273 | 1,610 | 106 | 553 | 8398.8 |
| **2017** | 914,773 | 1,485 | 72 | 616 | 12705.2 |
| **2018** | 1,003,636 | 1,744 | 104 | 575.5 | 9650.3 |
| **2019** | 1,036,318 | 1,660 | 92 | 624.3 | 11264.3 |
| **2020** | 1,044,581 | 1,674 | 89 | 624 | 11736.9 |
| The ratio was calculated by dividing the total number of drug administration days by serum level tests or TDM.  ^*^The ratio was rounded to the first decimal place. | | | | | |

Supplementary Table 18 Total number of drug administration days, number of serum level tests and therapeutic drug monitoring, and average drug administration days per patients who received serum level tests or therapeutic drug monitoring by year with phenytoin in Seoul National University Bundang Hospital

| **Seoul National University Bundang Hospital** | | | | | |
| --- | --- | --- | --- | --- | --- |
| **Phenytoin** | **Total number of Drug**  **administration days**  **(day)** | **Number of patients who performed serum level tests**  **(N)** | **Number of patients who performed TDM**  **(N)** | **Average drug administration days per patients who performed serum level test^*^**  **(day)** | **Average drug administration days per patients who performed TDM^*^ (day)** |
| **2007** | 41,527 | 174 | 39 | 238.7 | 1064.8 |
| **2008** | 39,777 | 174 | 48 | 228.6 | 828.7 |
| **2009** | 35,221 | 186 | 61 | 189.4 | 577.4 |
| **2010** | 36,357 | 216 | 85 | 168.3 | 427.7 |
| **2011** | 37,415 | 157 | 45 | 238.3 | 831.4 |
| **2012** | 38,063 | 156 | 37 | 244 | 1028.7 |
| **2013** | 39,484 | 175 | 42 | 225.6 | 940.1 |
| **2014** | 42,147 | 179 | 61 | 235.5 | 690.9 |
| **2015** | 37,138 | 178 | 44 | 208.6 | 844 |
| **2016** | 39,062 | 160 | 50 | 244.1 | 781.2 |
| **2017** | 30,849 | 134 | 26 | 230.2 | 1186.5 |
| **2018** | 38,367 | 98 | 13 | 391.5 | 2951.3 |
| **2019** | 38,918 | 63 | 8 | 617.7 | 4864.8 |
| **2020** | 20,374 | 39 | 1 | 522.4 | 20374 |
| The ratio was calculated by dividing the total number of drug administration days by serum level tests or TDM.  ^*^The ratio was rounded to the first decimal place. | | | | | |

Supplementary Table 19 Total number of drug administration days, number of serum level tests and therapeutic drug monitoring, and average drug administration days per patients who received serum level tests or therapeutic drug monitoring by year with carbamazepine in Seoul National University Bundang Hospital

| **Seoul National University Bundang Hospital** | | | | | |
| --- | --- | --- | --- | --- | --- |
| **Carbamazepine** | **Total number of Drug**  **administration days**  **(day)** | **Number of patients who performed serum level tests**  **(N)** | **Number of patients who performed TDM**  **(N)** | **Average drug administration days per patients who performed serum level test^*^**  **(day)** | **Average drug administration days per patients who performed TDM^*^ (day)** |
| **2007** | 139,401 | 289 | 21 | 482.4 | 6638.1 |
| **2008** | 160,993 | 309 | 18 | 521 | 8944.1 |
| **2009** | 155,004 | 315 | 15 | 492.1 | 10333.6 |
| **2010** | 150,809 | 340 | 19 | 443.6 | 7937.3 |
| **2011** | 146,625 | 272 | 13 | 539.1 | 11278.8 |
| **2012** | 145,158 | 259 | 12 | 560.5 | 12096.5 |
| **2013** | 136,561 | 228 | 6 | 599 | 22760.2 |
| **2014** | 147,640 | 226 | 11 | 653.3 | 13421.8 |
| **2015** | 141,099 | 224 | 9 | 629.9 | 15677.7 |
| **2016** | 139,886 | 202 | 10 | 692.5 | 13988.6 |
| **2017** | 143,190 | 217 | 5 | 659.9 | 28638 |
| **2018** | 140,281 | 179 | 1 | 783.7 | 140281 |
| **2019** | 143,071 | 188 | 4 | 761 | 35767.8 |
| **2020** | 149,898 | 184 | 3 | 814.7 | 49966 |
| The ratio was calculated by dividing the total number of drug administration days by serum level tests or TDM.  ^*^The ratio was rounded to the first decimal place. | | | | | |

Supplementary Table 20 Total number of drug administration days, number of serum level tests and therapeutic drug monitoring, and average drug administration days per patients who received serum level tests or therapeutic drug monitoring by year with phenobarbital in Seoul National University Bundang Hospital

| **Seoul National University Bundang Hospital** | | | | | |
| --- | --- | --- | --- | --- | --- |
| **Phenobarbital** | **Total number of Drug**  **administration days**  **(day)** | **Number of patients who performed serum level tests**  **(N)** | **Number of patients who performed TDM**  **(N)** | **Average drug administration days per patients who performed serum level test^*^**  **(day)** | **Average drug administration days per patients who performed TDM^*^ (day)** |
| **2007** | 16,593 | 47 | 11 | 353 | 1508.5 |
| **2008** | 14,387 | 49 | 5 | 293.6 | 2877.4 |
| **2009** | 17,721 | 45 | 7 | 393.8 | 2531.6 |
| **2010** | 27,916 | 51 | 7 | 547.4 | 3988 |
| **2011** | 25,504 | 42 | 9 | 607.2 | 2833.8 |
| **2012** | 20,457 | 52 | 7 | 393.4 | 2922.4 |
| **2013** | 15,693 | 56 | 8 | 280.2 | 1961.6 |
| **2014** | 17,781 | 53 | 7 | 335.5 | 2540.1 |
| **2015** | 21,386 | 70 | 7 | 305.5 | 3055.1 |
| **2016** | 23,107 | 60 | 8 | 385.1 | 2888.4 |
| **2017** | 21,280 | 68 | 4 | 312.9 | 5320 |
| **2018** | 17,220 | 63 | 3 | 273.3 | 5740 |
| **2019** | 9,660 | 62 | 5 | 155.8 | 1932 |
| **2020** | 12,995 | 57 | 4 | 228 | 3248.8 |
| The ratio was calculated by dividing the total number of drug administration days by serum level tests or TDM.  ^*^The ratio was rounded to the first decimal place. | | | | | |

Supplementary Table 21 Total number of drug administration days, number of serum level tests and therapeutic drug monitoring, and average drug administration days per patients who received serum level tests or therapeutic drug monitoring by year with digoxin in Seoul National University Bundang Hospital

| **Seoul National University Bundang Hospital** | | | | | |
| --- | --- | --- | --- | --- | --- |
| **Digoxin** | **Total number of Drug**  **administration days**  **(day)** | **Number of patients who performed serum level tests**  **(N)** | **Number of patients who performed TDM**  **(N)** | **Average drug administration days per patients who performed serum level test^*^**  **(day)** | **Average drug administration days per patients who performed TDM^*^ (day)** |
| **2007** | 162,106 | 272 | 189 | 596 | 857.7 |
| **2008** | 180,117 | 244 | 178 | 738.2 | 1011.9 |
| **2009** | 196,676 | 282 | 203 | 697.4 | 968.8 |
| **2010** | 201,200 | 312 | 229 | 644.9 | 878.6 |
| **2011** | 203,101 | 283 | 218 | 717.7 | 931.7 |
| **2012** | 201,560 | 287 | 238 | 702.3 | 846.9 |
| **2013** | 217,708 | 259 | 217 | 840.6 | 1003.3 |
| **2014** | 223,760 | 298 | 238 | 750.9 | 940.2 |
| **2015** | 232,240 | 294 | 235 | 789.9 | 988.3 |
| **2016** | 256,663 | 306 | 254 | 838.8 | 1010.5 |
| **2017** | 252,892 | 259 | 204 | 976.4 | 1239.7 |
| **2018** | 243,625 | 276 | 227 | 882.7 | 1073.2 |
| **2019** | 229,349 | 261 | 205 | 878.7 | 1118.8 |
| **2020** | 215,604 | 219 | 186 | 984.5 | 1159.2 |
| The ratio was calculated by dividing the total number of drug administration days by serum level tests or TDM.  ^*^The ratio was rounded to the first decimal place. | | | | | |

Supplementary Table 22 Total number of drug administration days, number of serum level tests and therapeutic drug monitoring, and average drug administration days per patients who received serum level tests or therapeutic drug monitoring by year with theophylline in Seoul National University Bundang Hospital

| **Seoul National University Bundang Hospital** | | | | | |
| --- | --- | --- | --- | --- | --- |
| **Theophylline** | **Total number of Drug**  **administration days**  **(day)** | **Number of patients who performed serum level tests**  **(N)** | **Number of patients who performed TDM**  **(N)** | **Average drug administration days per patients who performed serum level test^*^**  **(day)** | **Average drug administration days per patients who performed TDM^*^ (day)** |
| **2007** | 99,725 | 158 | 128 | 631.2 | 779.1 |
| **2008** | 93,455 | 152 | 125 | 614.8 | 747.6 |
| **2009** | 111,551 | 158 | 146 | 706 | 764 |
| **2010** | 128,989 | 138 | 129 | 934.7 | 999.9 |
| **2011** | 106,145 | 98 | 95 | 1083.1 | 1117.3 |
| **2012** | 81,991 | 91 | 85 | 901 | 964.6 |
| **2013** | 78,235 | 100 | 89 | 782.4 | 879 |
| **2014** | 70,268 | 57 | 52 | 1232.8 | 1351.3 |
| **2015** | 51,939 | 38 | 32 | 1366.8 | 1623.1 |
| **2016** | 51,837 | 20 | 19 | 2591.9 | 2728.3 |
| **2017** | 51,918 | 22 | 18 | 2359.9 | 2884.3 |
| **2018** | 53,861 | 20 | 17 | 2693.1 | 3168.3 |
| **2019** | 44,517 | 22 | 21 | 2023.5 | 2119.9 |
| **2020** | 30,873 | 23 | 22 | 1342.3 | 1403.3 |
| The ratio was calculated by dividing the total number of drug administration days by serum level tests or TDM.  ^*^The ratio was rounded to the first decimal place. | | | | | |

Supplementary Table 23 Total number of drug administration days, number of serum level tests and therapeutic drug monitoring, and average drug administration days per patients who received serum level tests or therapeutic drug monitoring by year with lithium in Seoul National University Bundang Hospital

| **Seoul National University Bundang Hospital** | | | | | |
| --- | --- | --- | --- | --- | --- |
| **Lithium** | **Total number of Drug**  **administration days**  **(day)** | **Number of patients who performed serum level tests**  **(N)** | **Number of patients who performed TDM**  **(N)** | **Average drug administration days per patients who performed serum level test^*^**  **(day)** | **Average drug administration days per patients who performed TDM^*^ (day)** |
| **2007** | 103,100 | 414 | - | 779.1 | - |
| **2008** | 101,911 | 362 | - | 747.6 | - |
| **2009** | 102,808 | 336 | - | 764 | - |
| **2010** | 117,867 | 353 | - | 999.9 | - |
| **2011** | 117,197 | 367 | - | 1117.3 | - |
| **2012** | 133,372 | 444 | - | 964.6 | - |
| **2013** | 144,998 | 350 | - | 879 | - |
| **2014** | 150,141 | 415 | - | 1351.3 | - |
| **2015** | 162,715 | 434 | - | 1623.1 | - |
| **2016** | 182,023 | 491 | - | 2728.3 | - |
| **2017** | 176,954 | 403 | - | 2884.3 | - |
| **2018** | 211,723 | 567 | - | 3168.3 | - |
| **2019** | 296,729 | 852 | - | 2119.9 | - |
| **2020** | 344,895 | 832 | - | 1403.3 | - |
| The ratio was calculated by dividing the total number of drug administration days by serum level tests or TDM.  ^*^The ratio was rounded to the first decimal place. | | | | | |
